# Smart Carbon Fiber-Reinforced Polymer Composites for Damage Sensing and On-Line Structural Health Monitoring Applications

**DOI:** 10.3390/polym16192698

**Published:** 2024-09-24

**Authors:** Cláudia Lopes, Andreia Araújo, Fernando Silva, Panagiotis-Nektarios Pappas, Stefania Termine, Aikaterini-Flora A. Trompeta, Costas A. Charitidis, Carla Martins, Sacha T. Mould, Raquel M. Santos

**Affiliations:** 1 Physics Centre of Minho and Porto Universities (CF-UM-UP), University of Minho, 4710-057 Braga, Portugal; 2 LaPMET—Laboratory of Physics for Materials and Emergent Technologies, University of Minho, 4710-057 Braga, Portugal; 3 INEGI—Institute of Science and Innovation in Mechanical and Industrial Engineering, 4200-465 Porto, Portugal; aaraujo@inegi.up.pt (A.A.); fdsilva@inegi.up.pt (F.S.); 4 FORTH/ICE-HT Institute of Chemical Engineering Sciences, Foundation of Research and Technology Hellas, 26504 Patras, Greece; ppappas@iceht.forth.gr; 5 R-NANO—Research Lab of Advanced, Composite, Nano-Materials and Nanotechnology, Materials Science and Engineering Department, School of Chemical Engineering, National Technical University of Athens, 15773 Athens, Greece; stermine@chemeng.ntua.gr (S.T.); ktrompeta@chemeng.ntua.gr (A.-F.A.T.); charitidis@chemeng.ntua.gr (C.A.C.); 6 IPC—Institute for Polymers and Composites, University of Minho, Campus de Azurém, 4800-058 Guimarães, Portugal; cmartins@dep.uminho.pt (C.M.); sacha.tm@dep.uminho.pt (S.T.M.); 7 LAETA—Associated Laboratory for Energy, Transports and Aerospace, 4200-465 Porto, Portugal

**Keywords:** carbon fiber-reinforced polymer composites, nanomaterials, self-sensing, damage detection, structural health monitoring, nanocomposites, geometric features

## Abstract

High electrical conductivity, along with high piezoresistive sensitivity and stretchability, are crucial for designing and developing nanocomposite strain sensors for damage sensing and on-line structural health monitoring of smart carbon fiber-reinforced polymer (CFRP) composites. In this study, the influence of the geometric features and loadings of carbon-based nanomaterials, including reduced graphene oxide (rGO) or carbon nanofibers (CNFs), on the tunable strain-sensing capabilities of epoxy-based nanocomposites was investigated. This work revealed distinct strain-sensing behavior and sensitivities (gauge factor, GF) depending on both factors. The highest GF values were attained with 0.13 wt.% of rGO at various strains. The stability and reproducibility of the most promising self-sensing nanocomposites were also evaluated through ten stretching/relaxing cycles, and a distinct behavior was observed. While the deformation of the conductive network formed by rGO proved to be predominantly elastic and reversible, nanocomposite sensors containing 0.714 wt.% of CNFs showed that new conductive pathways were established between neighboring CNFs. Based on the best results, formulations were selected for the manufacturing of pre-impregnated materials and related smart CFRP composites. Digital image correlation was synchronized with electrical resistance variation to study the strain-sensing capabilities of modified CFRP composites (at 90° orientation). Promising results were achieved through the incorporation of CNFs since they are able to form new conductive pathways and penetrate between micrometer-sized fibers.

## 1. Introduction

Damage tolerance is still a major challenge in the design, manufacturing, and lifetime of traditional carbon fiber-reinforced polymer (CFRP) composites, particularly because of the strict requirements for safety and operation reliability in highly demanding applications, such as aerospace and racing. Several approaches have been widely investigated for CFRP damage detection and structural health monitoring, including non-destructive testing (NDT), such as acoustic emission, ultrasonics, X-ray computed tomography, and infrared thermography, as well as embedded sensors as optical fiber Bragg gratings (FBGs) and lead zirconate titanate (PZT, piezoelectric), among others [1,2,3]. Despite their numerous advantages, most of these methodologies not only require additional sensors, complex equipment, intricate assembly, and significant costs [4,5], but they can also interfere with the overall mechanical performance of composite materials [6,7]. For instance, the main drawbacks of inserting extrinsic sensors in CFRPs concern their size (much larger than the size of the reinforcing fibers) and elastic property mismatches. Inserting FBG-based sensors leads to the formation of an eye-shaped pocket that can trigger cracks through the layers perpendicularly to the reinforcement fibers. A similar outcome is expected for composites with embedded PZT, in particular, because of its extremely brittle ceramic nature [8]. Moreover, since experiments are typically performed offline, real-time or -operation evaluation of the material’s structural integrity is restricted.

In this context, self-sensing strategies have been explored as a promising approach for evaluating CFRP composite structures. The intrinsic conductivity of carbon fibers (CFs) enables the investigation of fiber-dominated failure modes that typically occur near the end-of-life (EoL) of these materials [9]. This can be achieved by investigating changes in electrical resistance (Δ*R*, where *R* is the electrical resistance) while applying different mechanical stimuli (strain, *ϵ*, or stress, *σ*) [10]. Although CFRP composites are electrically conductive in both longitudinal and through-thickness directions, their conductive behavior is strongly dependent on the fiber volume fraction, fiber conductivity, applied loading, laminate geometry/sequence, etc. [3]. For instance, while the longitudinal electrical conductivity in CFRPs is primarily governed by the continuous conduction along the fibers, the lower conductivity in the transverse direction is promoted by fiber–fiber contacts within an insulating matrix [11,12].

The emergence of multifunctional carbon-based nanomaterials with large specific surface areas and distinct geometric features, such as one-dimensional (1-D) carbon nanotubes (CNTs) and nanofibers (CNFs), and two-dimensional (2-D) graphene and its derivatives, boosted the development of electrically conductive polymeric matrices. These matrices are capable of detecting early failure modes surrounding carbon fibers, such as microcrack, debonding, and/or delamination [13]. Nevertheless, precise morphological control of the conductive networks formed during the preparation of nanocomposites is crucial, as it directly influences the final CFRP composite properties and strain-sensing capacity [14]. This can be achieved not only by optimizing the concentration of nanofiller (ranging from 0.01 to 10 wt.%) but also by ensuring optimal dispersion within the polymeric matrix [15].

For a successful dispersion of carbon-based nanomaterials within a polymeric matrix, strong hydrodynamic stresses, extensional flows, and appropriate residence times are essential. Despite other important technical details, the operating conditions must be carefully optimized depending on the geometric features of carbon-based nanomaterials, as their dispersion is governed by different mechanisms and kinetics [16]. For example, I. Alig et al. [17] studied the dispersion of multiwall carbon nanotubes (MWCNTs), revealing that agglomerates were typically dispersed by rupture and erosion mechanisms, which usually occur simultaneously. While at low mixing speeds, dispersion was dominated by both mechanisms, rupture governance increased with increasing mixing speed. The authors also established a critical correlation between electrical conductivity and the dispersion level attained. However, the dispersion mechanism of 2-D layered structures is even more challenging owing to the strong interaction and small spacing between layers, hindering the intercalation of the polymeric chains and their full exfoliation [18].

Therefore, achieving an effective dispersion of nanomaterials within matrix-rich regions to form percolating pathways without compromising the structural performance of the composite while maintaining high piezoresistive sensitivity is a key objective for smart CFRP composites with self-sensing applications. At the matrix level, Wang et al. [14] prepared epoxy nanocomposites with different loadings of CNFs and reported high values of gauge factors for nanocomposites at 0.29 vol. % and 1.16 vol. % concentrations, thus validating their possible use for self-sensing applications. At the CFRP composite level, E. T. Thostenson and T.-S. Chou [19] investigated the use of CNTs as in situ sensors for real-time monitoring of damage accumulation during cyclic loading of glass fiber-reinforced polymer (GFRP) composite materials. Their study revealed substantial hysteresis in the electrical response due to the formation and opening/closing of cracks behavior, providing valuable insights into the extent of damage. Additionally, Gao et al. [20] used CNTs to achieve the successful real-time monitoring of structural damage patterns and growth during incremental cyclic tensile loading tests in modified CFRPs. To our knowledge, the usage of CNFs as strain sensors in CFRP is limited [21]. Bhandari et al. [22] evaluated the piezoresistivity of hybridized CNM-modified glass or carbon FRP. CNT–CNF GFRP composites showed the highest gauge factor values compared with the other hybrid materials. However, the results for the modified CFRPs showed deterioration of the gauge factor, possibly due to the high filler loading and particle dispersion state [22]. Concurrently, most of the investigations dealing with the use of rGO are performed on GFRP where the fibers are coated with a nanofiller [23,24]. Noora Alahmed et al. [25] compared the piezoresistivity of rGO-coated glass fiber-based sensors in dry form and GFRP, denoting a higher gauge factor for the dry material (without matrix).

In this work, the influence of the geometric features of 2-D layered reduced graphene oxide (rGO) and 1-D tubular CNFs on the electrical percolation threshold and strain-sensing capabilities of epoxy-based nanocomposites will be presented. Special attention will be devoted to the dispersion optimization of the developed formulations to ensure high electrical conductivity at low contents, without compromising the elongation at break of the material. This will be achieved through the utilization of a three-roll mill, which is a well-established and scalable methodology [26]. Since the available investigations in CFRPs using modified epoxy matrices with these types of fillers are very limited, the most promising formulations from this work will be used for the production of unidirectional (UD) prepreg materials. After conversion into composite laminates, the strain-sensing capabilities of the modified CFRP composites will be investigated to assess the potential use of CNFs and rGO in damage sensing and on-line structural monitoring.

## 2. Materials and Methods

### 2.1. Materials

A conventional system used in aeronautic applications, based on diglycidyl ether of bisphenol A (Araldite LY 556^®^), was employed as a pre-polymer matrix in combination with (i) accelerator DY 070^®^ and hardener HY 906^®^ for the preparation of dog-bone shaped samples used in electro-mechanical characterization and volumetric electrical properties and (ii) aradur 1571^®^, accelerator 1573^®^, and hardener XB 3430^®^ for the manufacturing of unidirectional (UD) pre-impregnated materials. Two systems were used since the former has a sufficiently high viscosity, which prevents sample preparation without the presence of voids, even when different strategies are applied (e.g., pressure and/or temperature). The properties of each component and the mix ratio per 100 g of pre-polymer are summarized in Table 1, according to the supplier’s datasheet, aiming to attain a glass transition temperature (Tg) higher than 120 °C. All components were purchased from Huntsman Corporation^®^, Advanced Materials, Frankfurt, Germany.

Two-dimensional layered reduced graphene oxide (rGO) was synthesized by exfoliation of natural graphite (NGS, Naturgraphit GmbH^®^, Leinburg, Germany), using a modified version of the Hummers method with an extra pre-oxidation step, followed by the final oxidation, where graphene oxide (GO) was collected [27]. Furthermore, GO flakes were heated to 1323.15 K to promote their reduction, giving rise to reduced graphene oxide.

One-dimensional tubular CNFs were produced by thermal chemical vapor deposition (T-CVD), using nickel as a catalyst. Specifically, the supported catalyst approach was used for current synthesis, according to which the catalyst in powder form was placed on an inert substrate (Si wafer or ceramic boat) in the reactor prior to the initiation of the reaction. Acetylene (60 sccm, 99.9% Evripos Gases, Dima, Aikaterini, & Co., Ritsona, Greece) was used as the carbon source, while N_2_ (240 sccm, N50 50 lt/200 bar, Evripos Gases) was used as the inert and carrier gas. The flow rate of the gases was controlled manually, according to the indications of the digital mass flow meters (FMA-A2000, Omega, Norwalk, CT, USA) installed prior to the inlet of the system. The reaction took place at 1 023.15 K, for 1 to 4 h, in order to collect the necessary quantity of CNFs in powder form.

**Figure 1:**
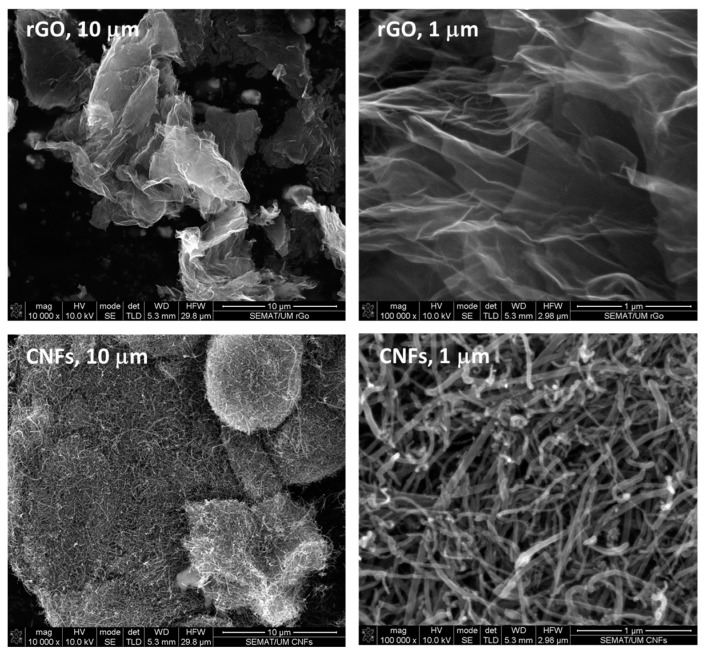
SEM images of 2-D layered rGO and 1-D tubular CNFs used in this study at different magnifications (10,000× and 100,000×), respectively.

The morphology of both rGO and CNFs was analyzed by scanning electron microscopy (SEM) using a NanoSEM-FEI Nova 200 (FEG/SEM) microscope (from FEI Company, part of Thermo Fisher Scientific, Hillsboro, OR, USA) with an integrated EDAX-Pegasus X4M (EDS/EBSD) apparatus, at magnifications of 10,000× and 100,000× (Figure 1). Raman spectroscopy was carried out in backscattering configuration with an inVia Reflex microscope using a solid-state laser (λ_0_ = 532 nm) (from Renishaw plc, Gloucestershire, UK) as the excitation source. The laser beam was focused onto the samples by means of a 100× magnification lens. The analysis of the scattered beam was performed on a 250 mm focal length spectrometer along with 1800 lines/mm. For comparison purposes, the luminescence background was subtracted by cubic spline interpolation, while spectral deconvolution was carried out by non-linear least squares fitting of the Raman peaks to a mixture of Lorentzian and Gaussian line shapes. The frequency shifts were calibrated by an internal Si reference.

According to the SEM images, thin and sharp flakes, which were difficult to image individually, were clearly identified for rGO. The crumpled and folded flakes, comprising only a few layers of very thin crystals, are promoted by the bidimensional membrane structure becoming thermodynamically stable via bending [27]. On the other hand, the produced CNFs exhibited lengths in the order of 1 μm, and the diameter varied from 15 to 50 nm. Branches were also observed in most fibers, which can be mainly described as straight structures with a needle-like geometry.

**Figure 2:**
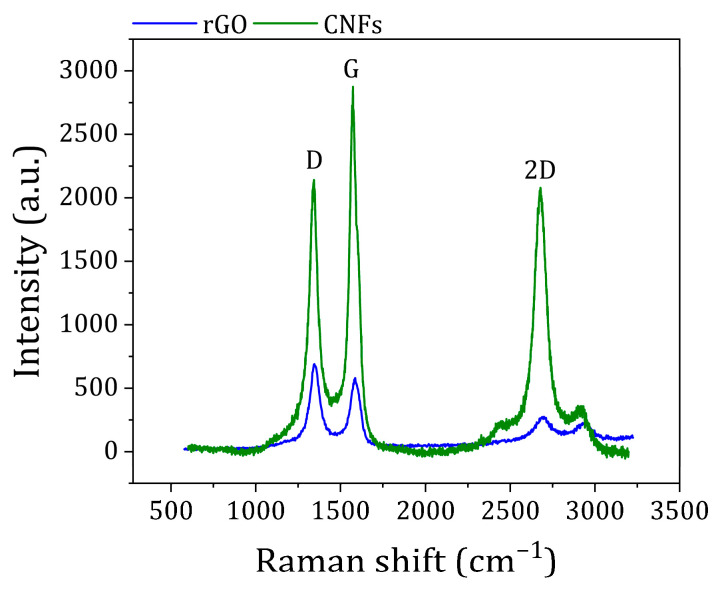
Raman spectra of synthesized rGO and CNFs.

Raman spectroscopy is essential for the structural characterization of carbon-based nanomaterials since it allows for the distinction between different forms of carbon and monolayers from bi-, tri-, and multi-layer forms of rGO, among others [28]. The Raman spectra of rGO and CNFs are presented in Figure 2.

The first-order tangential G band was observed near 1576 cm^−1^, which is related to the stretching bond of all pairs of sp^2^ carbon atoms in both rings and chains, corresponding to an ideal graphitic lattice vibration with E_2g_ symmetry [29,30]. The D band, another first-order band characteristic of disordered graphite, was observed near 1374 cm^−1^. The D band originates on a double-resonance process involving an inelastic scattering by a phonon and an elastic scattering by a defect, and it is strongly enhanced when structural disorder increases [31]. Therefore, the second-order Raman scattering of the G′ band (or 2-D) and the D+G band were also observed and centered at 2680 and 2910 cm^−1^, respectively. In addition, the ratio of areas of D and G bands, A_D_/A_G_, is commonly used to determine changes in defect concentration. The Raman spectrum of rGO revealed a more intense D band in comparison with the G band, suggesting a significant disorder in the graphene lattice induced by the harsh conditions applied to effectively exfoliate graphite [27]. On the other hand, the ID/TG ratio determined for CNFs showed that is equal to 2.39, which indicates a highly distorted internal wall structure, representative for CNFs.

Commercially available carbon fibers (CFs), T700SC12K-50C-PP^®^ (from Toray Carbon Magic Co., Ltd.^®^, Otsu, Japan), with a standard tensile modulus (231 GPa), a high tensile strength (5090 MPa), and a density of 1.8 g·cm^−3^, were used as advanced reinforcements for developing UD prepreg materials and related CFRP composites.

### 2.2. Preparation and Characterization of Epoxy-Based Nanocomposites

Epoxy-based nanocomposites containing rGO or CNFs, at different loadings, were prepared using an intensive mixer (EXAKT 80^®^, from EXAKT Technologies Inc., Oklahoma City, OK, USA) at a controlled gap distance between rolls, angular velocity, and number of passages per cycle. Table 2 presents the dispersion conditions applied to develop and optimize the nanocomposites.

The agglomerates size and number in the epoxy-based nanocomposites containing fillers with different geometric features were evaluated by light optical microscopy (LOM) in transmission mode. Thin sections with a thickness of 5 μm were cut using a microtome Leitz 1401 (from Leitz, Wetzlar, Germany) with a glass knife at an angle of 45° and placed between glass slides. A BH2 Olympus^®^ microscope (from Olympus Corporation, Tokyo, Japan) with a 1.6× ocular and 20× objective magnification (total area of analysis of 3.5 × 10^5^ µm^2^) combined with a digital camera Leica DFC 280^®^ (from Leica Microsystems, Wetzlar, Germany) was used. ImageJ 1.52e^®^ software was used to analyze 20 micrographs for each formulation and to determine the area ratio (A_r_, %) and the number of agglomerates per unit area (N, mm^−2^).

Immediately after dispersion, the suspensions were mixed with the accelerator DY 070^®^ and hardener HY 906^®^ using a mechanical stirrer for 10 min at 1000 rpm. The final suspensions were poured into a dog-bone shaped silicone mold and cured at 393.15 K for 2 h, and a post-curing step was carried out at 453.15 K for additional 2 h. The dog-bone shaped specimens were used to investigate their piezoresistive behavior during uniaxial and cyclic tensile tests.

The electrical percolation threshold (Φ_C_) of the epoxy-based nanocomposites was obtained from the volume electrical conductivity using the two-oribes method. The samples were prepared by sputter deposition of two parallel gold/palladium (Au/Pd) electrodes with 5 mm diameter at both ends of the sample using a SC502 sputter coater (from Bio-Rad Laboratories, Hercules, CA, USA). The resistivity of the samples was assessed by applying voltage steps from −10 to +10 V, while the electrical current was measured with an automated picoammeter/voltage source Keithley 487. The electrical conductivity (σ) was determined by the slope of the I−V curves attending to the geometrical parameters, according to Equation (1) [32]:

(1) $$\sigma =\frac{1}{\rho }=\frac{I}{V}\frac{L}{A},$$

where *I* is the electrical current, *V* is the voltage, *L* is the distance located at the top of the sample, and *A* is the electrodes area.

The piezoresistive behavior of the epoxy-based nanocomposites was assessed by measuring the instantaneous resistance variation during tensile tests, at room temperature (RT), in agreement with both ASTM D257 [33] and ISO 527 [34]. The dog-bone shaped specimens were electrically insulated with sandpaper to avoid leakage currents through the electrodes and reduce the stress concentration from the gripping during tensile tests. For the uniaxial and cyclic tensile loadings, an INSTRON 5900R^®^ (from INSTRON, Norwood, MA, USA) was used with a crosshead speed of 1 mm·min^−1^ and a load cell of 5 kN. The nanocomposite self-sensing capabilities was evaluated by using Equation (2) [35]:

(2) GF=∆RR0ε,

where ε is the instantaneous strain and ΔR is the difference between the strained gauge factor (GF) resistance and unstrained resistance (*R*_0_) of the specimen at strain ε, and the resistance of the non-strained specimen R_0_, expressed as Equation (3) [35]:

(3) $$∆R=R\left(\varepsilon \right)-R_{0}$$

After a detailed analysis of the strain sensitivity, the nanocomposites with the most promising piezoresistive behavior were submitted to additional cyclic mechanical loadings, up to a maximum displacement of 1.5 mm, to evaluate their stability and reproducibility. Thus, six additional dog-bone shaped test specimens of each formulation were submitted to ten load/unload combined cycles in real time, up to a critical strain of 1.3% (from elastic to plastic transition). The experimental procedure to measure the electrical resistance during the combined cycles was kept unchanged. 

### 2.3. Manufacturing and Characterization of UD Prepreg Materials and Composites

UD prepreg materials were prepared using a laboratory drumwinder under optimized processing conditions (summarized in Table 3) to ensure a final fiber volume fraction (FVF) of approximately 60%.

The FVF of each prepreg material was determined according to ASTM D3529 M [36]. It is noteworthy that higher shear viscosities of modified suspensions with rGO, introduced minor variations in the FVF of the produced prepreg materials. Moreover, the surface mass (SM), and fiber areal weight (FAW) obtained for each formulation are summarized in Table 4.

Afterward, UD prepreg sheets with 270 × 270 × 2 mm^3^ dimensions were hand lay-up and consolidated in an autoclave at 393.15 K and 3.5 bar for 2 h. CFRP specimens containing 24 plies (2 mm thickness) were produced for transversal tensile tests (90° orientation, in which matrix-dominated failure modes predominate), according to the standard ASTM D3039/D3039M [37], aiming to evaluate the electrical response promoted by the matrix under loading conditions.

The mechanical failure detection of unmodified and modified CFRP composites with rGO or CNFs during loading conditions was assessed using the piezoresistive sensitivity of the selected formulations combined with digital image correlation (DIC) and electrical resistance variation. Before measurements, a speckle pattern over the specimen composite surface was created for DIC, consisting of black dots randomly distributed over a white background by means of spray painting. Then, onto the back side of the specimen, four pairs of electrodes were directly applied using silver paint, spaced by 3.5 mm, following the same approach used for the epoxy-based nanocomposites. Note that the DIC results obtained were originally computed in pixels (using a computer vision-based system) and then converted into millimeters. Because of the potentialities of this full-field contactless optical method technique, during each experimental tensile testing, the displacements were measured in very short periods (10 screenshot/s). 

## 3. Results and Discussion

### 3.1. Morphology and Dispersion State Characterization

**Figure 3:**
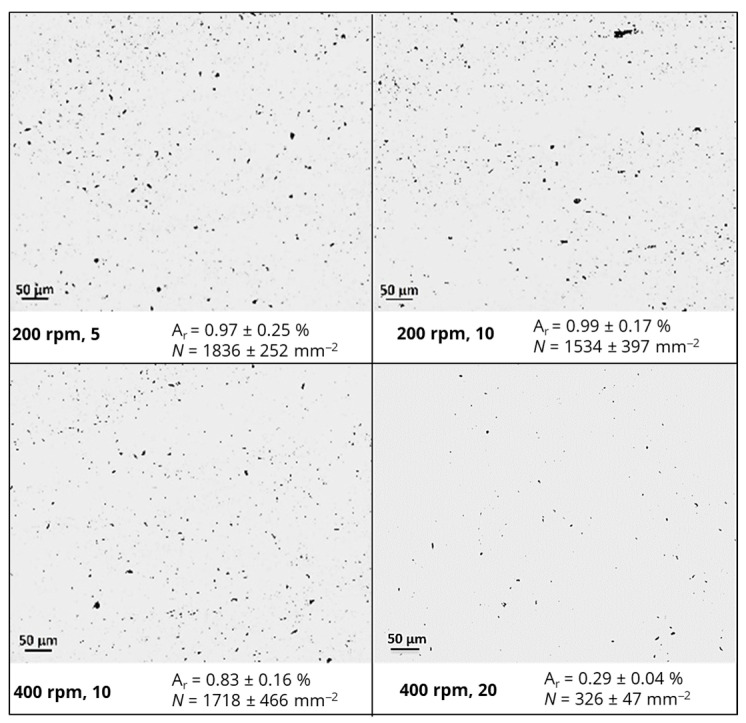
Optical micrographs illustrating the morphology and dispersion state of epoxy-based nanocomposites containing rGO.

**Figure 4:**
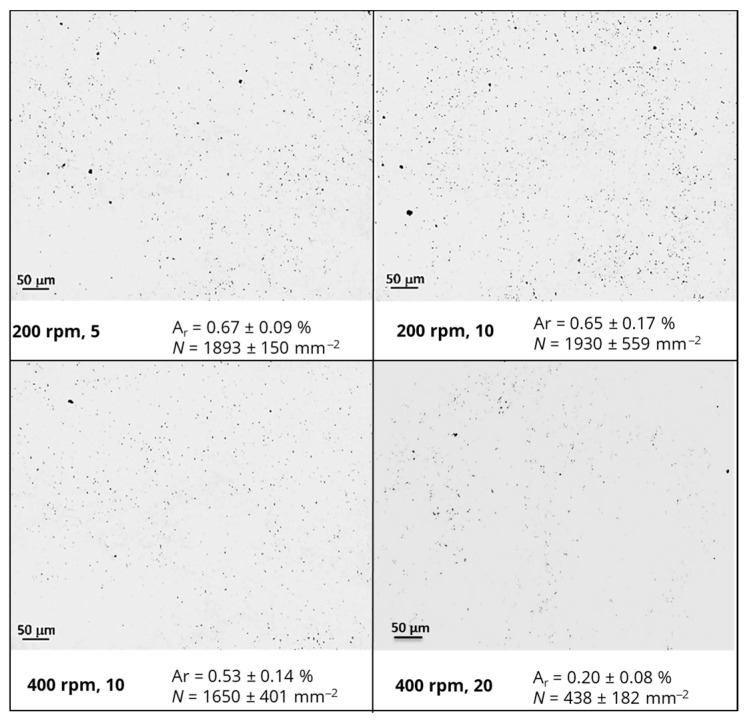
Optical micrographs illustrating the morphology and dispersion state of epoxy-based nanocomposites containing CNFs.

The dispersion state of nanofillers within polymeric matrices plays a crucial role in the design and development of electrically conductive nanocomposites for strain-sensing applications [16]. The morphology and extent of dispersion of modified epoxy resin with rGO or CNFs, prepared by using an intensive mixer under different conditions, were evaluated by LOM. The results are depicted in Figure 3 and Figure 4, respectively.

A similar trend was observed for epoxy-based nanocomposites containing rGO or CNFs, i.e., a significant decrease in the size and number of agglomerates per unit area when a longer mixing time and higher angular velocities were applied. In fact, the residence time influenced the infiltration of the polymer into the initial agglomerates and the mixing energy incorporated in the system, while higher shear stresses promoted the fragmentation of nanofiller agglomerates. Indeed, *N* for nanocomposites containing rGO was reduced from 1836 ± 252 to 326 ± 47 mm^−2^, while for those modified with CNFs, *N* decreased from 1893 ± 150 to 438 ± 182 mm^−2^ when 400 rpm and 20 passages per each cycle were applied. At a constant concentration and hydrodynamic stresses, the results also evidenced that a better dispersion was attained for rGO, with improvements of 82% compared with 77% for nanocomposites containing CNFs. This difference is attributed to the lower values of density and cohesive strength present on the initial rGO agglomerates, as postulated by Manas-Zloczower and co-authors [38]. Moreover, interactions between rGO, containing residual functional groups from the exfoliation of the natural graphite, and the epoxy matrix (polar polymer) were more favorable than those for CNFs, influencing the dispersion kinetics and extent.

### 3.2. Electrical Conductivity and Strain-Sensing Capabilities of Epoxy-Based Nanocomposites

**Figure 5:**
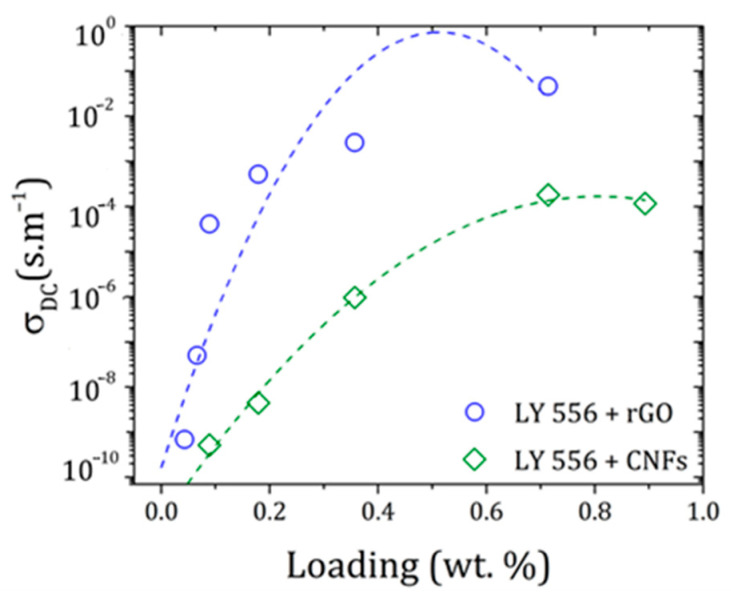
Volumetric electrical properties as a function of rGO and CNF loadings.

Since high electrically conductive materials are desirable for strain-sensing applications, without compromising their ductility, the influence of different rGO or CNFs loadings on the volumetric electrical properties of the epoxy resin was investigated. The results are depicted in Figure 5.

The electrical resistivity of 1.2 × 10^−11^ S∙m^−1^ obtained for the epoxy resin confirmed its insulating nature. At ultralow rGO contents (0.089 wt.%), the electrical conductivity increased up to 4 × 10^−5^ S∙m^−1^, reaching 5 × 10^−2^ S∙m^−1^ with 0.714 wt.% of incorporation. These results evidence that a good rGO dispersion was attained, allowing the formation of conductive pathways within the matrix. Regarding epoxy-based nanocomposites containing CNFs, a similar insulator-to-semi-conductor transition was observed at higher loadings. In fact, a maximum of 2 × 10^−4^ S∙m^−1^ was obtained for a 0.714 wt.% concentration of CNFs, suggesting that poor connections between nanofibers were formed.

Although the dispersion conditions were adjusted considering the geometric features of each carbon-based nanomaterial (1-D tubular CNFs and 2-D layered rGO), the results pointed out that other parameters apart from dispersion have a significant impact on the final electrical properties of nanocomposites. These include the quality of the nanofiller structure, aspect ratio, agglomerate cohesive strength, and electrical resistance at the nanofiller/polymer interface, among others [17].

Therefore, formulations containing 0.089 of rGO or 0.714 of CNFs were selected to investigate the strain-sensing capabilities of the epoxy-based nanocomposites based on piezoresistivity. This consisted of evaluating how the nanofiller state of percolation affects the materials’ electrical resistance responses to an external stress/strain stimulus.

Here, two additional formulations with 0.130 wt.% of rGO and 1.000 wt.% of CNFs were also prepared and characterized, since nanocomposites with filler contents above Φ_C_ are expected to exhibit a high piezoresistive sensitivity because of inherent loose connections between fillers [35]. At higher loadings of rGO and CNFs, although an electrical conductivity plateau is reached, both conductive network and piezoresistive sensing become more stable because of the formation of relatively denser spatial pathways [39].

**Figure 6:**
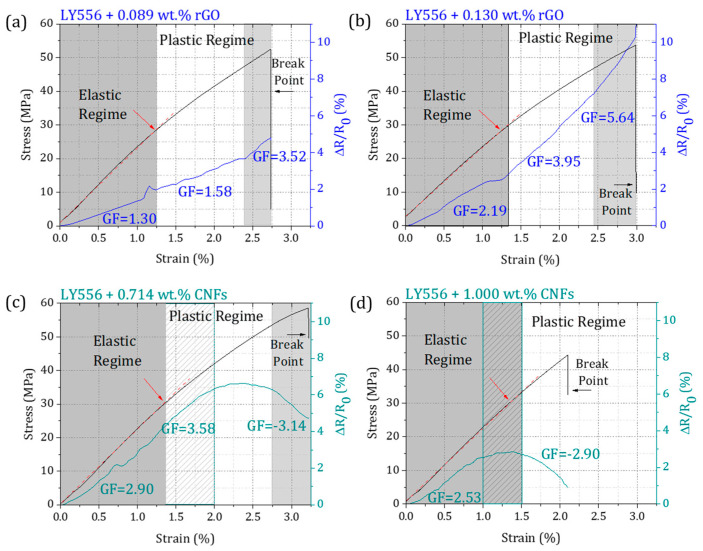
The piezoresistive response of the representative epoxy-based nanocomposites prepared with different formulations.

The piezoresistive response of epoxy-based nanocomposites during tensile tests is shown in Figure 6. To facilitate interpretation, the elastic and plastic regimes were identified for each representative specimen by the deviation of the tangent (red dash line) from the initial linear portion of the stress–strain curve. In addition, the gauge factor (GF) was calculated to evaluate the sensitivity of the nanocomposite strain sensors at different strains.

A distinct strain-sensing behavior was systematically observed for nanocomposites containing 2-D layered rGO or 1-D tubular CNFs. Regardless of the geometric features or nanofiller loadings, the ΔR/R_0_–strain curves showed three different regions of linear fitting, indicating varying strain sensitivities during mechanical deformation. The in situ electrical resistance of epoxy/rGO increased monotonically and with a steeper increment at strains up to 2.5 or 3%, indicating the breakage of the conductive network. On the other hand, in nanocomposites containing CNFs, the electrical resistance increased up to a certain strain and then decreased. This behavior can be explained by (i) the mixed sensing model (the tunneling effect and conduction path), as already reported for carbon-based nanomaterials having similar tubular features, and (ii) the formation of new or alternative conductive networks between neighboring CNFs, where contact resistance overtakes tunneling resistance, enhancing conductivity (negative GF values) [40].

The sensitivity analysis of the strain sensor showed that the incorporation of higher loadings of rGO led to the highest GF values (5.64), owing to the formation of a greater number of conductive pathways. Conversely, the nanocomposites with 1.000 wt.% of CNFs evidenced poor strain-sensitive sensitivity, characterized by the turning point in the electrical conductivity (negative GF). This is likely related to the presence of a higher number or larger CNF agglomerates, especially at high filler loadings. Moreover, the results evidenced that 0.714 wt.% of CNFs exhibited comparable sensitivity to 0.130 wt.% of rGO for lower strains (up to 1%), whereas rGO was more suitable for sensing higher strains (up to 2.5–3%).

**Figure 7:**
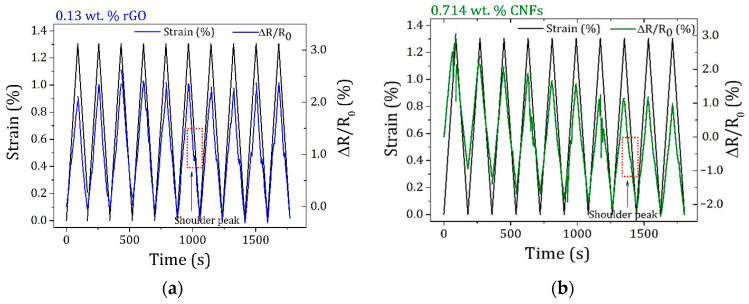
Representative loading profiles under 10 stretching/relaxing cycles up to 1.3% strain for epoxy-based nanocomposites containing (**a**) 0.13 wt.% of rGO and (**b**) 0.714 wt.% of CNFs.

All epoxy-based nanocomposites presented an inhomogeneous electrical response to the stress distribution, either by an increment or signal change in GF, allowing for the prediction of the elastic regime and mechanical failure. In this context, the nanocomposite sensors with the most promising tunable strain sensitivity were submitted to loading profiles to investigate their stability and reproducibility. The results are displayed in Figure 7.

For both nanocomposite sensors, an increase in ΔR/R_0_ with increasing strain was observed because of the reduction in contact points between nanofillers, while the electrical resistance decreased with decreasing strain, promoted by the reconstruction of conductive networks.

During cyclic loading, different behaviors were observed for nanofillers with distinct geometric features. For nanocomposites containing 0.13 wt.% of 2-D layered rGO, the electrical resistance variation remained quasi-stabilized after the third cycle and the conductive network showed a notable preservation after 10 stretching/relaxing cycles. For those modified with 1-D tubular CNFs, a noticeable decrease in maximum relative resistance was found. According to the literature [41], this behavior is associated with the formation of additional conductive pathways through the breakdown and subsequent formation of interfaces between the CNFs and the insulating phases of the polymer.

**Figure 8:**
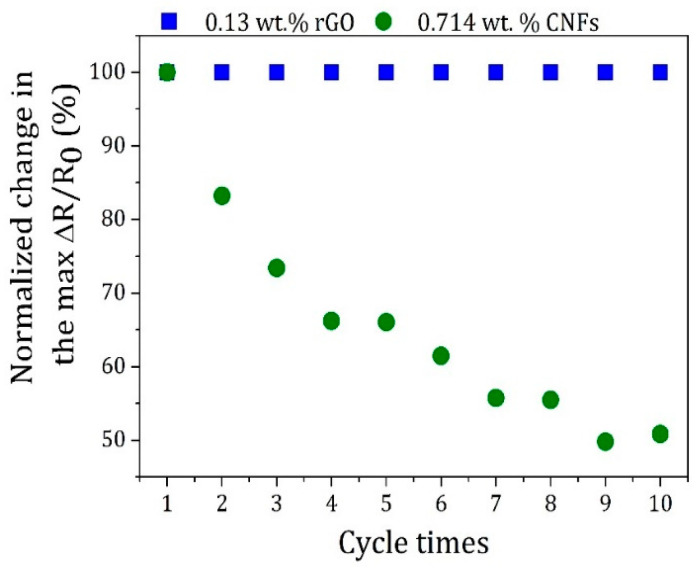
Normalized change in the maximum relative resistance variation (ΔR/R_0_, %) for nanocomposite sensors containing 0.13 wt.% of rGO and 0.714 wt.% of CNFs during 10 stretching/relaxing cycles.

For both types of nanocomposite sensors, competition between the destruction and reorganization of conductive pathways occurred, as evidenced by the common shoulder peaks observed during dynamic loadings. To further understand the rearrangements that occurred on the conductive networks, the maximum ΔR/R_0_ of each cycle was normalized to the first one during 10 stretching/relaxing cycles (Figure 8).

The results showed that the deformation of the conductive network formed by adding 0.130 wt.% of rGO was predominantly elastic, and its destruction during stretching cycles can be assumed as reversible. In contrast, for nanocomposites containing 0.714 wt.% of CNFs, the electrical conductivity increased with the number of dynamic tensile cycles, which can be attributed to the reconstruction of new conductive paths or conduction mechanisms, as already mentioned.

### 3.3. Electrical Conductivity and Strain-Sensing Capabilities of CFRP Composites

The aforementioned discussion showed that rGO and CNF nanocomposites have distinct strain-sensing capabilities; therefore, both were investigated as potential candidates for developing smart CFRP composites for self-sensing applications. Here, epoxy-based formulations containing 0.130 wt.% of rGO or 0.714 wt.% of CNFs were reinforced with continuous carbon fibers to produce UD prepreg materials and related CFRP composites.

**Figure 9:**
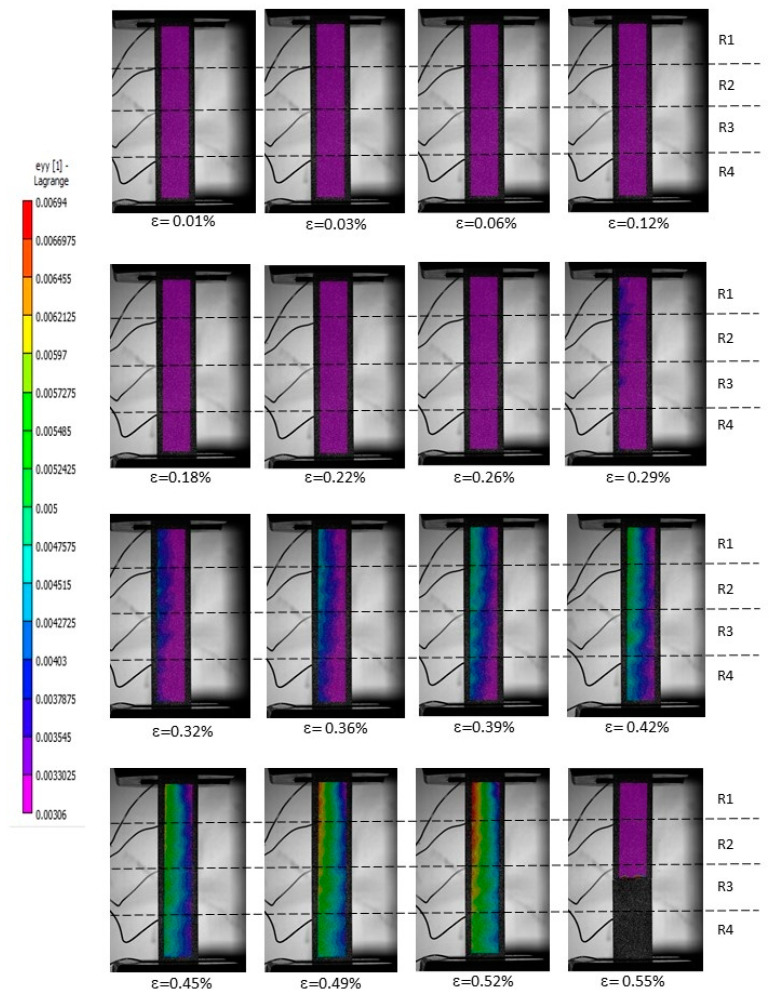
The strain full-field images captured by DIC, showing the damage evolution stages from 0.01 to 0.55% strain, where tensile failure occurred during mechanical testing, for a representative unmodified CFRP composite specimen. R1–R4 are the locations where electrodes combined to strain gauges were used for measurements.

**Figure 10:**
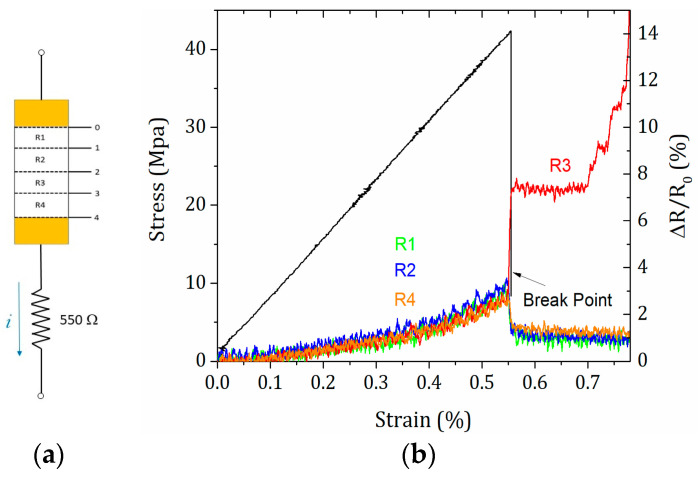
(**a**) Representative instrumentation of an unmodified CFRP composite specimen. (**b**) In situ mapping recorded at R1–R4.

The piezoresistive sensitivity of unmodified and modified CFRP composites was studied through the synchronization of DIC with electrical resistance variation, at different zones upon loading conditions. The DIC strain full-field images showing the stages of damage evolution until tensile failure, along with in situ mapping (recorded at R1–R4 electrodes combined with strain gauges, which were placed locally on the surface of specimens) are depicted in Figure 9 and Figure 10, for a representative unmodified CFRP composite. The first image without loading was set as a reference, and displacements in the thickness direction were neglected since they are small.

Unmodified CFRP composites are electrically conductive even in the transverse direction, in which the epoxy matrix with an insulating nature plays a major role. This behavior is promoted by fiber–fiber contacts and, consequently, the formation of conductive pathways [11]. Based on DIC measurements (Figure 9), the damage pattern and progress (location and width) for this representative unmodified CFRP specimen were identified, showing an increasing number of areas with higher strain values (especially after the ninth image or global strains higher than 0.32%), suggesting the formation and propagation of microcracks and defects within the composite structure. Overall, from R1 to R4, a uniformed longitudinal stress distribution was observed, with slight fluctuations between 0.0014 and 0.00026 for global strains between 0.32% and 0.42%. These fluctuations, more concentrated on the left side of the image (green region), are a direct consequence of the extra surface tension provided by electrodes. Mechanical failure occurred at the R3 zone (ε = 0.55%), leading to a pronounced electrical variation. During loading, all zones showed a similar behavior, i.e., the resistance increased linearly proportional to strain (Figure 9) At ε = 0.55%, where the R1, R2, and R4 values fell because of stress suppression.

Despite the different types of damage that CFRP composites can experience under loading, matrix structural failure is one of the most common because of its brittle behavior and much lower strength than CFs. In this context, the sensing of the matrix behavior is of paramount importance in predicting the potential mechanical failure of the CFRP composite structure in earlier stages.

**Figure 11:**
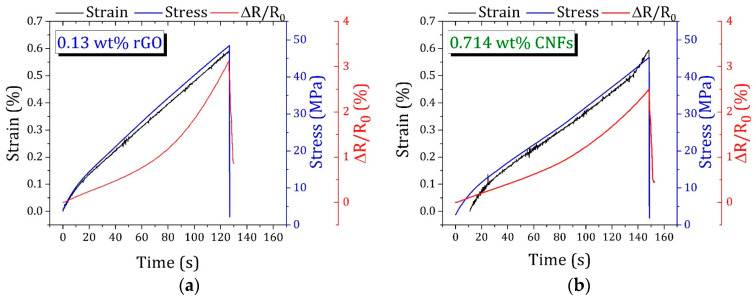
In situ sensing of representative modified CFRP composite samples with (**a**) 0.13 wt.% of rGO and (**b**) 0.714 wt.% of CNFs.

Figure 11 presents the in situ sensing of the modified CFRP composites with rGO and CNFs.

It is challenging to decouple the electrical signals of the embedded carbon-based networks from the highly conductive CFs [42]. The following three distinct stages with tunable sensitivities were identified for CFRP composites containing 0.13 wt.% of rGO: (i) a linear stage until 0.1% of strain, (ii) a non-linear stage up to 0.4%, and (iii) an abrupt increase until the ultimate failure, showing the possibility of using CFRP composites as strain sensors. Interestingly, this trend tended to disappear for modified CFRP composites with 0.714 wt.% of CNFs, where a highly linear relationship was established between strain deformation and electrical resistance, attesting to the ability of CNFs to establish new conductive networks under loading. Thostenson and C. Tsu-Wei [43] investigated nanomaterials with similar tubular features and showed that 1-D carbon fibers with nanometer-sized diameters have the ability to penetrate through micrometer-sized fibers and form a conductive percolating network in the polymeric matrix.

These results showed the potential of using smart CFRP composites based on self-sensing nanocomposites for early damage detection and structural health monitoring.

## 4. Conclusions

The first part of this study focused on developing epoxy-based nanocomposites with high electrical conductivity and strain-sensing capacities, using low contents of carbon-based nanomaterials. To achieve this, the dispersion conditions were optimized, considering the geometric features of 2-D layered rGO and 1-D tubular CNFs. The experimental results can be summarized as follows:

(1) To promote the fragmentation of rGO or CNF agglomerates, high hydrodynamic shear stresses and long residence times are required (400 rpm and 20 passages per cycle).
(2) An insulator-to-semi-conductive transition was attained with the incorporation of either rGO or CNFs, reaching maximum electrical conductivities at 0.714 wt.% of rGO or CNFs (5 5 × 10^−2^ or 2 × 10^−4^ S∙m^−1^, respectively).
(3) The strain-sensing capabilities of epoxy-based nanocomposites are strongly dependent on the nanofiller concentration and geometric features. Although high electrical conductivity is required, the ductility of the final material must not be compromised.
(4) Nanocomposites containing 0.13 wt.% of rGO showed remarkable sensing stability and reproducibility, a crucial behavior for real applications of strain sensors. An interesting behavior was found for modified counterparts with CNFs, where the electrical conductivity increased with an increasing number of tensile cycles, associated with the formation of new or alternative conductive pathways.

The last part of this work aimed to investigate the influence of self-sensing nanocomposite sensors, based on rGO or CNFs, for early detection of damage or on-line structural health monitoring in smart CFRP composites.

(5) DIC measurements allowed us to identify the damage pattern and progress, including location and width.
(6) The electrical signal variation synchronized with DIC also showed three distinct stages, having tunable sensitivities, for modified CFRP composites with 0.13 wt.% of rGO.
(7) A different behavior was found for CFRP composites containing CNFs, where a more linear relationship was established between strain deformation and electrical resistance.

In addition, this study revealed that this not straightforward to transfer the sensing capabilities from modified matrices with carbon-based nanomaterials to CFRP composites, especially for damage sensing or on-line structural monitoring applications. This is mainly due to the high electrical conductivity of CFs and related CFRP composites, even in the 90° direction where the polymeric matrix plays a major role. In fact, the decoupling of electrical signals provided by embedded carbon-based networks or CFs is technically challenging. To address these challenges, several strategies as diagnostic tools to provide better insights into damage initiation and propagation in smart CFRP composites should be explored (e.g., by combining DIC with surface acoustic PZT sensors to determine the electromechanical impedance as a function of different displacement steps).

## Figures and Tables

**Table 1:** Properties of each component and the mix ratio per 100 g of pre-polymer used for the electro-mechanical characterization and manufacturing of UD prepreg materials.

Component	Viscosity at 298.15 K (mPa.s)	Density (g·cm^−3^)	Mix Ratio (g)
Araldite LY 556^®^	10,000–12,000	1.15–1.20	100
Aradur 1571^®^	28,000–40,000	1.20	23
Accelerator 1573^®^	60,000–90,000	1.08	5
Hardener XB 3403^®^	5–20	1.00	12
Accelerator DY 070^®^	≤50	0.95–1.05	2
Hardener HY 906^®^	175–350	1.20–1.25	95

**Table 2:** Dispersion conditions applied to develop and optimize nanocomposites.

Dispersion Conditions	Velocity (rpm)	Number of Passages per Each Cycle	Maximum Nominal Shear Rate (s^−1^)
200 rpm, 5	200	5	220,000
200 rpm, 10	200	10	
400 rpm, 10	400	10	440,000
400 rpm, 20	400	20	

**Table 3:** Conditions used to manufacture UD prepreg materials.

Fiber Tension (N)	Winder Speed (rpm)	Squeeze Rolls Distance (cm)	Pitch (cm)	Bath Temperature (K)
1.2	2	0.50	0.7	298.15

**Table 4:** Characterization of the produced UD prepreg materials with unmodified and modified resin with rGO or CNFs.

Code	SM (g·m^−2^)	FAW (g·m^−2^)	FVF (%)
Unmodified prepreg	151.83 ± 4.12	105.47 ± 1.18	60.5 ± 1.7
rGO	149.20 ± 4.69	105.68 ± 2.22	61.9 ± 2.0
CNFs	151.08 ± 2.56	104.94 ± 2.28	60.3 ± 0.7

## Data Availability

The original contributions presented in this study are included in the article, further inquiries can be directed to the corresponding authors.
